# Learning Family: Concept, Measurement and the Effect on Individuals’ Behaviors

**DOI:** 10.3390/bs14111061

**Published:** 2024-11-07

**Authors:** Ming Kong, Yahua Lu

**Affiliations:** 1 Antai College of Economics and Management, Shanghai Jiao Tong University, Shanghai 200030, China; 2 School of Education, Shanghai Jiao Tong University, Shanghai 200240, China; luyahua000@sjtu.edu.cn

**Keywords:** learning family, bioecological system theory, concepts, characteristic dimension, individuals’ behavior

## Abstract

A learning society cannot be built without each family playing its role. The learning family integrates the modern education concept and the actual needs of family education. However, scholars are still exploring the concept, structure, and measuring tools of the learning family. Based on the bioecological system theory, this study explored the concept and characteristic dimension of the learning family and verified the effect of the learning family through cross-population samples. The results of this study showed that, first, the learning family involves a process of mutual influence on the part of family members, who view the enhancement of comprehensive literacy as their core goal and can continuously accumulate knowledge and improve skills from the internal and external environments of the family through learning support and learning involvement, thereby achieving common growth, the main structure of which includes four characterizing dimensions of learning involvement, learning effectiveness, learning support and continuous learning. Second, the developed learning family scale has good reliability and validity. Third, learning families have significant positive effects on individual creativity, innovative behavior, prosocial behavior, proactive behavior, work performance, academic achievement, and science literacy. This study not only deepens our understanding of the importance of family learning and family education but also contributes to exploring the influence mechanisms underlying learning families on individual psychology and behavior in the future.

## 1. Introduction

As the starting point and foundation of education, the family is not only the primary place for individual growth but also an important field for shaping individual character and transmitting social values; furthermore, the quality of family learning and family education is directly related to an individual’s ability to become a high-quality talent who can meet the needs of their society in the future [1]. Moreover, family learning and family education constitute a complex and multilayered structural system; however, previous studies have usually regarded family learning and family education simply as concepts and modes of parental parenting [2], mainly from the perspectives of social learning theory and attachment theory, and sought to explore the impact of parental parenting on individual development [3,4]; this research perspective has thus been relatively limited. The structure, life and internal and external relationships of the modern family constitute the network that shapes family learning and family education. Family learning and family education should shift from a focus on simply answering the questions of what parents should do and how they can do it to an emphasis on the family as an environment that influences the development of the individual based on the particular kinds of life forms and activities it involves [5]. As a sign of the times for the development of family education, a learning family is an important basic element of a learning society, providing a new path through which family members can better adapt and contribute to society.

One’s family has a long-term and decisive influence on a person and plays an important foundational role in their talent training [6]. However, previous studies have mainly focused on the influence of social and school education on individual growth, and the role of family learning and family education in individual growth needs to be further explored [7]. Due to the ongoing changes in the structure and function of the family, problems arising from a lack of family education frequently emerge, and some parents, in the process of raising their children, face the problem of raising their children without educating them, or educating them inappropriately, which to a certain extent hinders the comprehensive development of the individual [8]. Therefore, family learning and family education are not only related to the quality of an individual’s growth environment but also to his or her potential for future development, especially the impact of a learning family on individual growth.

The construction of a learning family involves not only the parenting style of the parents but also the family learning life that family members experience and shape jointly as they grow [9]. The bioecological system theory provides a strong theoretical foundation for understanding the relationships between individuals and their surroundings [10]. Moreover, the process–person–context–time (PPCT) model of the bioecological system theory emphasizes the interactions among processes, people, the environment and time [11]. However, previous studies have utilized this theory mainly to explore the impacts of the microsystem, mesosystem, exosystem and macrosystem on individual development, thereby treating the family as a subsystem that affects individual development [12,13]; accordingly, they have not yet fully explored the complexity of family learning and family education or the relationships between these notions and a variety of environmental factors. Moreover, the emphasis of previous research on the personal and temporal elements of the process of theoretical evolution has been ignored. Therefore, it is necessary to re-examine the learning family in terms of the life process, the family learning environment and the individual development process.

At present, few empirical studies have examined the learning family, leading to its relatively limited interpretability and guidance of reality, with the greatest obstacle being the lack of research results on the development and testing of the learning family scale through standardized procedures, which has led to people still being confused about the basic problem of what the learning family entails. Additionally, it is not clear whether learning families affect individual behavior. Therefore, this study aimed to clarify the concept of the learning family and its characteristic dimensions, to develop a measurement instrument with good reliability and validity for the learning family, and to explore its effect on individual behavior.

This study aims to contribute to the literature in the following ways. First, based on the bioecological system theory, this study constructed a theoretical model of the learning family and revealed the concept of the learning family and its characteristic dimensions. Applying the PPCT model of the bioecological system theory to the study of family learning not only expands the application boundaries of the bioecological system theory but also provides a new theoretical perspective for understanding learning families in depth. Second, based on a rigorous scale development process, this study developed a learning family scale with good reliability and validity, which provides a foundation for future researchers to explore learning families. Third, this study validated the effect of the learning family on individuals in three groups—adolescents, college students, and on-the-job social personnel—and revealed the stability of the learning family scale across contexts.

## 2. Study 1: A Qualitative Study of Learning Family

Study 1 elucidated the concept of the learning family and its characteristic dimensions from theoretical and descriptive perspectives. Based on the characteristic dimensions of the learning family extracted from the theory, an open-ended questionnaire survey was adopted to collect the participants’ understandings and perceptions of the learning family and to develop the items of the learning family measurement questionnaire, laying the foundation for exploring the structure of the learning family.

### 2.1. The Concept and Characteristic Dimension of Learning Family

#### 2.1.1. Concept of Learning Family

At the beginning of the 21st century, in the context of advocacy for the construction of a learning society, the concept of the learning family also began to gradually appear in the public view [14]. The learning family was derived from the concept of the learning organization. Since the family is the most common and smallest form of organization in society, knowledge sharing and interaction among family members often have an important impact on the growth of each family member and the quality of life of the whole family [15,16]. In contrast to learning organizations that focus on the orientation of organizational learning outcomes [17], learning families emphasize the cultivation of learning abilities, rather than just the learning of knowledge points [14].

In previous studies on learning families, scholars have mainly explored the concept of learning families from a capability perspective per the belief that this family model reflects the family’s ability to adapt to changes in the social environment and the efforts of family members to improve the family’s overall quality of life [18]. Moreover, the learning family is also regarded as a new type of family education, in which each family member is considered to be a subject of learning [16], and through the establishment of a common vision, family members can learn independently and interactively and share the results of their learning [15]. Additionally, Liao [19] believes that the learning family consists of two aspects—“learning family” and “family learning”—with “learning family” referring mainly to the creation of a material environment conducive to learning and “family learning” referring to the learning activities in which family members participate together. Although previous studies initially explored the learning patterns among family members in the learning family, few studies have explored the learning process in which these family members interact with each other.

The learning family is related to the home learning environment, but the two have different concerns in terms of concepts and structures. The home learning environment refers to the range of parenting behaviors that parents provide for their children, such as educational activities, resources, and learning materials [20]. In previous research on home learning environments, individual home learning environments have been viewed primarily as the learning opportunities and conditions provided to the educated [21,22]. It is evident that previous research on the home learning environment has focused on the objective or material level of the environment and has not addressed the subjective or spiritual level. Therefore, this study will explore people’s perceptions of the learning family and its specific manifestations.

#### 2.1.2. Characteristic Dimensions of Learning Family

The learning family should be understood not only as a collection of family members’ learning ability but also in terms of the influence of systemic factors beyond the individual. The bioecological system theory of Bronfenbrenner [23] notes that an individual’s development is nested within a series of interconnected systems and that an individual’s development is the result of surrounding its proximal and distal systems. Therefore, the learning family can be regarded as a family ecosystem, and the development of the learning family as the result of interaction and coevolution between family members and the ecosystem. Moreover, the PPCT model of the bioecological system theory has been used to build a mature and comprehensive theoretical framework by emphasizing the comprehensive effects of process, person, context, and time on individual development [11,24]. The focus of this model is individuals’ subjectivity, and it profoundly reveals the important role of the multilevel environmental system in their development, which provides powerful theoretical support for thoroughly analyzing the characteristic dimensions of the learning family.

Specifically, first, according to the PPCT model of the bioecological system theory, “process” refers to interactions that occur between individuals and others and can maintain a certain frequency of interactions. Among these, the persistent form of interaction between individuals in the immediate environment is called the proximal process, which is the main driver of individual development [25]. For learning families, the proximal process mainly refers to learning interactions among family members, a process that involves not only the sharing of knowledge and experience but also the promotion of innovation and the practical application of knowledge while acquiring it [26].

Second, “person” refers to some characteristics of the individual, including knowledge, ability and curiosity [11]. Bioecological system theory focuses on the active role of influencing individuals in their own development, recognizing that developing individuals are active actors whose growths are affected not only by their surrounding environment but also by the environment in which they live [27]. For learning families, the cultivation of family members’ learning abilities includes not only formal learning activities within the family but also informal learning in family life.

Third, the “context” consists of four systems from near to far, namely, the microsystem, mesosystem, exosystem and macrosystem, which together influence the psychological state of the individual at the center [25]. For the learning family, the microsystem is focused on the family learning environment, the mesosystem covers the connection between family and school, the exosystem involves the working environment of parents and the macrosystem contains the social environment that advocates for lifelong learning and an increasingly developed networked information environment. All of these are multilevel environmental factors that invisibly affect individuals’ perceptions of the learning family.

Fourth, the bioecological system theory emphasizes the combination of time and environmental factors to examine the dynamic process of individual development, which is considered a continuous and changing process in which the duration and continuity of time play nonnegligible roles in the effect of interaction [11]. For learning families, continuous learning not only means that family members need to constantly update their knowledge systems and skill levels but also indicates that they can create opportunities for continuous learning [28]. In this process, family members should not only pay attention to their current learning progress but also have a long-term learning plan to cope with various challenges that may arise in the future.

**Figure 1:**
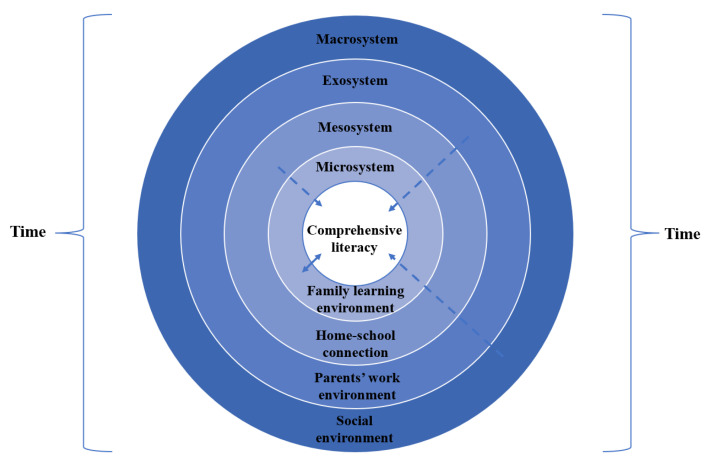
The bioecological system theoretical model of the learning family.

In summary, this study suggests that the learning family involves a process of mutual influence on the part of family members, who view the enhancement of comprehensive literacy as their core goal and can continuously accumulate knowledge and improve skills from the internal and external environments of the family through learning support and learning involvement, thereby achieving common growth. This process includes four characteristic dimensions: learning involvement, learning effectiveness, learning support and continuous learning. The theoretical model of the bioecological system of the learning family is presented in Figure 1, in which the dashed arrows pointing to the family members from the macrosystem, exosystem, and mesosystem indicate distal interactions, which indirectly affect the development of the family members, while the bidirectional arrows refer to proximal interactions, which directly affect the development of the family members in terms of learning.

### 2.2. Item Generation

#### 2.2.1. Participants

Participants were recruited through an online survey platform for generating descriptions of the learning family and were asked to describe in words the concept and characteristics of the learning family; this involved groups of college students, parents of primary and secondary schools, teachers, enterprise leaders, and employees. A total of 700 questionnaires were distributed, 600 questionnaires were recovered, and the questionnaires with too many blanks and inconsistent feedback content were removed, resulting in 500 valid questionnaires finally obtained, with an effective recovery rate of 83.33%. Of these participants, 36.00% were males and 64.00% were females, aged 18 to 57 years (*M* = 30.43, *SD* = 8.54).

#### 2.2.2. Results

First, a total of 5000 initial items were obtained from the valid questionnaires, and a total of 5179 items with a single meaning were extracted and retained by transforming the initial items into items with a single meaning and carrying out item extraction and information coding. Second, like items were combined. After combining items with identical descriptions and repetitive expressions, the number of items was reduced to 527, and items with similar content, expressed only in different ways, were combined a second time, resulting in a total of 108 items obtained after completion. Additionally, items with vague meanings and not closely related to the learning family were deleted by two experts, and a total of 50 items were obtained after this step was completed. Finally, based on the four characteristic dimensions obtained from the above theoretical framework, i.e., learning involvement, learning effectiveness, learning support, and continuous learning, and based on combining like items, we further summarized and generalized the items, and initially developed the items for the learning family measurement questionnaire. Then, two experts were invited to revise the questionnaire items that had been formed, and some items that were prone to ambiguity and did not conform to the dimensions were removed. At the same time, the readability of the questionnaire items was optimized, and the initial learning families scale containing 4 characteristic dimensions and 30 items was finally formed.

## 3. Study 2: Exploration of the Internal Structure of the Learning Family

To better validate the factor structure of the learning family and the validity of the measure, Study 2 further identified the characteristic dimensions of the learning family through an exploratory factor analysis of the initial questionnaire items of the learning family.

### 3.1. Participants

Questionnaires were distributed through an online survey platform; 683 questionnaires were distributed, 580 questionnaires were recovered, and after deleting the questionnaires that were answered too quickly, signified by choosing the same option for all the questions, answering with random regularity, or failing the attention test questions, the final valid sample was 553, and the recovery validity rate was 95.34%. Of these participants, 32.50% were males and 67.50% were females, aged 18 to 63 years old (*M* = 28.90, *SD* = 7.60), with education levels covering high school (2.53%), bachelor’s degrees (78.66%), master’s degrees (17.54%) and doctoral degrees (1.27%).

### 3.2. Measures

**Learning family**: We used the initial learning family scale developed in Study 1 with 30 items. The questionnaire was based on a 7-point Likert scale (1 = strongly disagree, 7 = strongly agree).

### 3.3. Results

#### 3.3.1. Item Analysis

First, the scores of each item in the learning family questionnaire were ranked, taking the top 27% of the total scores as the high group and the bottom 27% as the low group, and conducting independent samples *t*-tests on the scores of the high and low groups on each item. The results showed that all the items were significantly different in both the high and low groups, indicating that all the items had good discrimination. Second, it was seen after testing that the standard deviation of each item of the developed questionnaire was greater than 0.5, which indicated that the differentiation of the items of the questionnaire was good. Finally, the correlation analysis between the scores of each item and the total score revealed that all the items were correlated with the total score at a significance level of 0.01. Therefore, all the items were retained for further exploratory factor analysis.

#### 3.3.2. Exploratory Factor Analysis

Bartlett’s test of sphericity (*χ*^2^ = 3320.66, *df* = 120, *p* < 0.001) and the KMO test indicated suitability for exploratory factor analysis. We adopted a principal component analysis with the rotation method of maximum variance, with eigenvalues not less than 1 as the principle of factor extraction. Three types of items were removed: the commonality was less than 0.3, the absolute value of the load in each factor was less than 0.5 and the cross-loadings were greater than 0.3. The analysis was rerun for each removed item until no more items met any of the above conditions. Finally, the scale with 16 items distributed on 4 factors was obtained, and the cumulative percentage of variance for the 4 factors was 61.30%. The loadings of the items on the corresponding factors were in the range of 0.52 to 0.80, which better supported the four characteristic dimensions of Study 1 (see Table 1 for details).

## 4. Study 3: Reliability and Validity of the Learning Family

Based on the learning family measurement questionnaire obtained from Study 2, Study 3 further validated the characteristic dimensions of the questionnaire and its reliability and validity through a confirmatory factor analysis and a discriminant validity test.

### 4.1. Participants

Sample 1: For the confirmatory factor analysis, questionnaires were distributed through an online survey platform, 700 questionnaires were distributed and 500 questionnaires were recovered; after deleting the questionnaires that were answered too quickly, signified by choosing the same option for all the questions, answering with random regularity, or failing the attention test questions, the final valid sample was 485, and the recovery validity rate was 97.00%. Of these participants, 56.91% were males and 43.09% were females, aged 18 to 60 years old (*M* = 27.67, *SD* = 8.19), with education levels covering junior high school and below (0.41%), high school (4.74%), bachelor’s degrees (82.68%), master’s degrees (11.34%) and doctoral degrees (0.82%).

Sample 2: For the discriminant validity analysis, questionnaires were distributed through an online survey platform, 322 questionnaires were distributed and 250 questionnaires were recovered; after deleting the questionnaires that were answered too quickly, signified by choosing the same option for all the questions, answering with random regularity, or failing the attention test questions, the final valid sample was 217, and the recovery validity rate was 86.80%. Of these participants, 39.17% were males and 60.83% were females, aged 18 to 63 years old (*M* = 30.24, *SD* = 6.96), with education levels covering high school (0.92%), bachelor’s degrees (72.81%), master’s degrees (24.88%) and doctoral degrees (1.39%).

### 4.2. Measures

The participants in both Sample 1 and Sample 2 were required to complete the 16-item learning family scale retained after the exploratory factor analysis in Study 2, and the questionnaire was based on a 7-point Likert scale.

Following this, the participants in Sample 2 were also required to complete:

**Parental parenting styles:** We used the scale developed by Parker et al. [29] and asked the participants to respond by recalling memories of their parent–child interactions before the age of 16, with a total of 25 items; an example item is “Spoke to me with warm and friendly voice”. The scale was categorized into four dimensions: caring (α = 0.85), indifference/rejection (α = 0.79), overprotection (α = 0.76), and the allowance of autonomy and independence (α = 0.88), and the questionnaire was based on a 7-point Likert scale;

**Parental involvement:** We used a scale developed by Grolnick and Slowiaczek [30] with 3 items such as “My parents keep close track of how well I am doing in school” and the questionnaire was based on a 7-point Likert scale, with the Cronbach’α value of 0.78;

**Family functioning:** We used a scale developed by Epstein et al. [31] with 53 items, such as “We try to think of different ways to solve problems”, and the questionnaire was based on a 7-point Likert scale, with the Cronbach’α value of 0.94;

**Learning organization:** We used the scale developed by Goh and Richards [32] with 21 items such as “Managers in this organization frequently involve members of the organization in important decisions” and the questionnaire was based on a 7-point Likert scale, with the Cronbach’α value of 0.92.

### 4.3. Results

#### 4.3.1. Reliability Test

The α coefficients for each dimension of the Sample 1 learning family scale for learning involvement, learning effectiveness, learning support and continuous learning were 0.83, 0.82, 0.80 and 0.83, respectively, and the α coefficient for the total scale was 0.92. The α coefficients for each dimension of the Sample 2 learning family scale for learning involvement, learning effectiveness, learning support and continuous learning were 0.73, 0.77, 0.74 and 0.70, respectively, and the α coefficient for the total scale was 0.89. The reliability indicators were well-met.

#### 4.3.2. Confirmatory Factor Analysis

From the results of the confirmatory factor analysis in Table 2, it can be seen that the four-factor model was more advantageous in describing the internal structure of the learning family with better fitting results than the other factor models (*χ*^2^/*df* = 4.04, CFI = 0.92, TLI = 0.90, RMSEA = 0.08, SRMR = 0.05). Moreover, each item had high loadings on the corresponding factor, with standardized loadings ranging from 0.63 to 0.78, with *p* less than 0.001, and the measurement items were all valid. The average variance extraction (AVE) values for the four dimensions of learning involvement, learning effectiveness, learning support and continuous learning were 0.55, 0.53, 0.51 and 0.55, respectively, which were all greater than 0.50, and the aggregation validity was good. Additionally, the AVE square root values corresponding to the four factors were all greater than the correlation coefficients among the factors (see Table 3), indicating good discriminant validity.

#### 4.3.3. Discriminant Validity Test

To test whether there was good discriminant validity between the learning families and the other variables, this study examined parental parenting styles, parental involvement, family functioning and learning organization. In the family environment, parental parenting styles consist of a collection of attitudes, emotions and behavioral tendencies presented by parents in the process of educating and raising their children [33], while parental involvement is the degree to which parents are aware of and involved in their children’s academic fields [34]. Thus, the learning family, parental parenting styles and parental involvement are all theoretically individuals’ perceptions of their family education style. At the same time, as a comprehensive variable, family functioning covers family members’ emotional connection and behavioral regulation, as well as their ability to solve problems together [35]. To a certain extent, the learning family reflects the characteristics of family functioning. Additionally, learning organization tends to focus on learning as the key to continuously improving an organization [36], and since the concept of the learning family originates from learning organization, both include a commitment to achieving continuous progress and development through learning, despite differences in the level of application. These four variables are close to those of the learning family in terms of the theoretical constructs. Therefore, the method of Mathieu and Farr [37] was referenced for testing the discriminant validity of similar variables in this study.

First, we adopted Harman’s single-factor test to examine Sample 2 and found that there were 28 factors with eigenvalues greater than 1, and the cumulative variance explanation rate of the first precipitated factor was 28.26%, lower than the standard of 40%, which implied that the common method bias did not have a serious impact in this study. Second, discriminant validity analyses were conducted, and the results showed that the two-factor models were all superior to the one-factor models; specifically, the two-factor model of the learning family and parental parenting styles had better fitting results (*χ*^2^/*df* = 2.37, CFI = 0.98, TLI = 0.96, RMSEA = 0.08, SRMR = 0.02). The two-factor model of the learning family and parental involvement had better fitting results (*χ*^2^/*df* = 1.37, CFI = 0.99, TLI = 0.99, RMSEA = 0.04, SRMR = 0.02). The two-factor model of the learning family and family functioning had better fitting results (*χ*^2^/*df* = 2.42, CFI = 0.97, TLI = 0.95, RMSEA = 0.08, SRMR = 0.03). The two-factor model of the learning family and learning organization had better fitting results (*χ*^2^/*df* = 1.60, CFI = 0.99, TLI = 0.98, RMSEA = 0.05, SRMR = 0.02). Therefore, the above results indicate that the learning family is a different variable from parental parenting styles, parental involvement, family functioning, and learning organization, with good discriminant validity (see Table 4).

## 5. Study 4: Effectiveness of the Learning Family

### 5.1. Theoretical Hypothesis

The bioecological system theory emphasizes that the influence of the family environment on individuals is long-term and decisive [23], and that the learning family, as a specific family environment, has an important impact on individuals’ behavioral development and academic performance [1]. Specifically, creativity refers to an individual’s generation of novel and useful ideas in learning or work [38], while innovative behavior involves the embodiment of creativity in practical action and the behavior of individuals who generate new ideas or solutions in learning or work and try to put them into practice [39]. The members of a learning family are permanently in an environment with a strong learning atmosphere and support for innovation, which enables them to accumulate a richer stock of knowledge and to be more courageous in trying out new ideas and methods in the face of difficulties and challenges, which provides a solid foundation for stimulating individual creativity and innovative behavior [40]. Prosocial behaviors refer to individuals’ voluntary behaviors or tendencies that benefit others and society through social interactions, including sharing, cooperation, helping and caring [41]. The members of a learning family learn from each other and communicate equally, which can provide positive behavioral models for individuals, make them more concerned about the needs and feelings of others, and increase their willingness to share and cooperate with others [42], thus promoting the development of individual prosocial behavior. Proactive behavior refers to individuals’ spontaneous behavior with the goal of improving themselves and their environment, as a future orientation that involves challenging the status quo rather than passively adapting to existing conditions [43]. Learning families encourage family members to continuously learn and constantly improve their knowledge and skills so that they can face various challenges and opportunities in life with a more proactive attitude and maintain competitiveness and adaptability in the changing social environment [44], thus promoting individual proactive behaviors. Furthermore, the learning family can provide family members with more support in terms of time, emotion, experience and other resources, which facilitates individuals’ task commitment and duty fulfillment in the workplace [45], and thus improves their job performance.

Additionally, in a learning family, parents often have more input and support for their children’s academics and actively participate in their children’s education process by providing learning resources, guiding learning methods and encouraging them. As an important part of family social capital, these educational participation behaviors help to improve individuals’ academic performance [46,47]. Moreover, the learning family and individuals’ science literacy are closely linked. Science literacy refers to the understanding of basic scientific and technological constructs that are necessary for individuals to participate in social life [48]. Learning families not only emphasize children’s mastery of scientific knowledge but also pay more attention to stimulating their curiosity and encouraging them to actively explore and discover scientific knowledge, which helps to enhance individuals’ ability to apply scientific knowledge creatively in life situations [49], thus improving their science literacy. With all this considered, we hypothesize the following:

**Hypothesis 1.**  *Learning families are significantly and positively associated with individual creativity (H1a), innovative behaviors (H1b), prosocial behaviors (H1c), proactive behaviors (H1d), job performance (H1e), academic achievement (H1f) and science literacy (H1g).*

### 5.2. Method

#### 5.2.1. Participants

Sample 1: Used to examine the performance of individuals in different levels of learning family situations. Questionnaires were distributed to the college student population through an online survey platform, setting the occupation of the sample as college students, and a total of 263 questionnaires were distributed; after deleting the data that did not pass the attention test, 200 valid data were finally obtained. Of these participants, 32.50% were males and 67.50% were females, aged 18 to 30 years old (*M* = 21.68, *SD* = 2.18) and they comprised undergraduates (82.50%), master’s students (17.00%) and doctoral students (0.50%). The participants involved in the experiment were randomly assigned to an experimental group (100 participants) or a control group (100 participants).

Sample 2: Questionnaires were distributed to on-the-job social personnel through an online survey platform; setting the employment status of the sample to employed, a total of 264 questionnaires were distributed. After deleting the data that were answered with random regularity and those that failed to pass the attention test, 200 valid data were finally obtained. Of these participants, 30.00% were males and 70.00% were females, aged 21 to 58 years old (*M* = 31.88, *SD* = 5.63), with education levels covering high school (2.00%), bachelor’s degrees (67.50%), master’s degrees (29.50%) and doctoral degrees (1.00%), with the years of job tenure ranging from 1 to 35 (*M* = 7.57, *SD* = 5.11), with engagement in industries such as retail, manufacturing, IT, construction, finance, and energy.

Sample 3: A questionnaire survey was adopted to collect data with the cooperation of teachers and parents from several primary and secondary schools, covering several provinces, autonomous regions, and municipalities in China. Specifically, the teachers evaluated the students’ science literacy, prosocial behavior, and academic achievement, and the parents were asked to evaluate their child’s learning family, science literacy, parental educational expectations and family socioeconomic status, as well as their child’s demographic information. Of the 188 questionnaires distributed, 168 were recovered (recovery rate of 89.36%) and 145 were valid (effective rate of 86.31%). Thus, the data for Sample 3 consisted of paired data between the teachers and parents of the students. Of the 145 valid samples, 53.10% were females and 46.90% were males, with ages ranging from 6 to 17 years (*M* = 13.78, *SD* = 2.32), and grades ranging from 1 to 10 (*M* = 7.68, *SD* = 2.42).

#### 5.2.2. Measures

The participants in Sample 1, Sample 2, and Sample 3 were required to complete the 16-item learning family scale retained from the exploratory factor analysis in Study 2, and their questionnaires were based on a 7-point Likert scale. The Cronbach’α value of this scale in Sample 1 was 0.98; in Sample 2 it was 0.88; and in Sample 3 it was 0.92. We measured the following:

**Creativity:** We used the scale developed by Farmer et al. [38] with 4 items, such as “I can often seek new ideas and ways to solve problems”, and the questionnaire was based on a 7-point Likert scale. The Cronbach’s α value for this scale in Sample 1 was 0.95; in Sample 2 it was 0.82;

**Innovative behaviors:** We used the scale developed by Scott and Bruce [39] with 6 items, such as “I often generate creative ideas”, and the questionnaire was based on a 7-point Likert scale. The Cronbach’s α value for this scale in Sample 1 was 0.96; in Sample 2 it was 0.80;

**Prosocial behaviors:** We used the scale developed by Goodman [50] with 5 items, such as “I usually share with others (food, games, pens, etc.)”, and the questionnaire was based on a 7-point Likert scale. The Cronbach’s α value for this scale in Sample 1 was 0.96; in Sample 3 it was 0.96;

**Proactive behaviors:** We used the scale developed by Griffin et al. [51] with 9 items, such as “I will come up with ways of increasing efficiency within the organization”, and the questionnaire was based on a 7-point Likert scale. The Cronbach’s α value for this scale in Sample 1 was 0.97; in Sample 2 it was 0.86;

**Job performance:** We used the scale developed by Williams and Anderson [52] with 15 items, which was divided into three dimensions: in-role behavior, organizational citizenship behavior toward the organization, and organizational citizenship behavior that has a specific individual as the target. An example item was “I can fulfill responsibilities specified in the job description”, and the questionnaire was based on a 7-point Likert scale. The Cronbach’s α value for this scale in Sample 2 was 0.76;

**Academic achievement:** The teachers provided their students’ scores in Chinese, math, and English from their most recent final exam prior to this survey. According to Cheung and Pomerantz [46], for the students in each school, the original scores of the three subjects were standardized within the grade level, and then the standardized scores of the three subjects were added together as a measure of the student’s academic achievement;

**Science literacy:** We used the non-cognitive performance variables of PISA2015 on science literacy and selected instrumental motivation, interest in broad science topics, enjoyment of science, and science self-efficacy with reference to Zhao and Huang [53]. To reduce the error associated with social desirability, after recovering the data, science literacy was used as the mean of the results reported by the teachers and parents, and the questionnaire used a 7-point Likert scale. In Sample 3, the Cronbach’s α value of the teachers’ assessment of the students’ science literacy was 0.98, and the Cronbach’s α value of the parents’ assessment of their children’s science literacy was 0.97;

**Control variables:** According to previous studies, educational expectations have a significant impact on individuals’ behavior and academic performance [54], and family socioeconomic status affects individuals’ learning engagement and learning autonomy [55,56]. Therefore, self (or parental) educational expectations and family socioeconomic status were used as control variables in this study, where family socioeconomic status was statistically analyzed by transforming the scores of parental educational attainment, occupation, and monthly family income into standardized scores, respectively, with reference to relevant studies [57]. Additionally, this study also controlled for corresponding demographic variables.

#### 5.2.3. Procedure

A single-factor, two-level experiment was conducted on Sample 1, and the participants were randomly assigned to the high level of the learning family group (experimental group) and the low level of the learning family group (control group) to read the contextual materials of the high/low level of the learning family, respectively, which used typical scenarios that were mentioned more frequently in the data of Study 1 as the learning family contexts. After reading the contextual materials, the participants were asked to report on their perceived learning family and innovative behaviors, creative behaviors, prosocial behaviors, and proactive behaviors in the corresponding context. Additionally, the participants were asked to report their gender, age, education level, and self-educational expectations. Sample 2 adopted the questionnaire of the learning family, innovative behavior, creativity, proactive behavior, and job performance to measure the sample of the on-the-job social personnel. Sample 3 adopted the questionnaire of the learning family, science literacy, prosocial behavior, and academic achievement to measure the primary and secondary school teachers and parents, thus validating the relationship between the learning family and individual behaviors in different contexts.

### 5.3. Results

#### 5.3.1. Confirmatory Factor Analysis

As shown in Table 5, the five-factor model has better fitting results compared to the combination of factors in the other models (*χ*^2^/*df* = 1.90, CFI = 0.91, TLI = 0.90, RMSEA = 0.07, SRMR = 0.06), indicating good discriminant validity for the five main variables measured in Sample 2.

As shown in Table 6, the three-factor model has better fitting results compared to the combination of factors in the other models (*χ*^2^/*df* = 1.33, CFI = 0.99, TLI = 0.99, RMSEA = 0.05, SRMR = 0.04), indicating good discriminant validity for the three main variables measured in Sample 3.

#### 5.3.2. Common Method Bias

We adopted Harman’s single-factor test to examine Sample 2 and found that there were 19 factors with eigenvalues greater than 1, and the cumulative variance explanation rate of the first precipitated factor was 31.58%, lower than the standard of 40%, which implied that the common method bias did not have a serious impact in this study.

#### 5.3.3. Descriptive Statistics

Table 7 shows the means, standard deviations, and correlation coefficients among the variables involved in Sample 2. As expected, the learning family was significantly and positively correlated with innovative behavior (*r* = 0.70, *p* < 0.01), the learning family was significantly and positively correlated with creativity (*r* = 0.74, *p* < 0.01), the learning family was significantly and positively correlated with proactive behavior (*r* = 0.69, *p* < 0.01) and the learning family was significantly and positively correlated with job performance (*r* = 0.67, *p* < 0.01). These results provide a data basis for further research.

Table 8 shows the means, standard deviations, and correlation coefficients among the variables involved in Sample 3. As expected, the learning family was significantly and positively correlated with science literacy (*r* = 0.41, *p* < 0.01), the learning family was significantly and positively correlated with prosocial behavior (*r* = 0.23, *p* < 0.01), and the learning family was significantly and positively correlated with academic achievement (*r* = 0.18, *p* < 0.05). These results provide a data basis for further research.

#### 5.3.4. Hypothesis Testing

First, an independent samples *t*-test was conducted on Sample 1, and the results showed that the scores reported by the participants in the high-level learning family group (*M* = 6.05, *SD* = 0.56) were significantly higher than those in the low-level learning family group (*M* = 2.10, *SD* = 0.88), *t* (169) = 37.70, *p* < 0.001, indicating that the manipulation of the learning family in this study was valid.

As shown in Table 9, after controlling for the participants’ gender, age, education level, and self-educational expectation, the learning family had a significant positive correlation with innovative behaviors (*b* = 0.86, *p* < 0.001), the learning family had a significant positive correlation with creativity (*b* = 0.87, *p* < 0.001), the learning family had a significant positive correlation with prosocial behaviors (*b* = 0.85, *p* < 0.001), and the learning family had a significant positive correlation with proactive behaviors (*b* = 0.82, *p* < 0.001), indicating that the learning family influences the innovative behavior, creativity, prosocial behavior, and proactive behavior of college students.

Second, hypothesis testing was conducted for Sample 2. As shown in Table 10, after controlling for the participants’ gender, age, education level, job tenure, self-educational expectation, and family socioeconomic status, the learning family was significantly and positively associated with innovative behavior (*b* = 0.70, *p* < 0.001), the learning family was significantly and positively associated with creativity (*b* = 0.72, *p* < 0.001), the learning family was significantly and positively associated with proactive behaviors (*b* = 0.69, *p* < 0.001), and the learning family was significantly and positively associated with job performance (*b* = 0.68, *p* < 0.001), indicating that the learning family influences the innovative behavior, creativity, proactive behavior and job performance of the on-the-job social personnel.

Finally, hypothesis testing was conducted for Sample 3. As shown in Table 11, after controlling for the participants’ gender, age, grade, parental educational expectations and family socioeconomic status, the learning family was significantly and positively associated with students’ science literacy (*b* = 0.42, *p* < 0.001), the learning family was significantly and positively associated with prosocial behaviors (*b* = 0.23, *p* < 0.01), and the learning family was significantly and positively associated with academic achievement (*b* = 0.18, *p* < 0.05), indicating that the learning family influences the science literacy, prosocial behaviors and academic achievement of adolescents.

## 6. Discussion

This study progressively explored and validated the concept, characteristic dimensions and effects of the learning family through four sub-studies. Based on the bioecological system theory, Study 1 preliminarily proposed the concept and characteristic dimensions of the learning family, i.e., learning involvement, learning effectiveness, learning support and continuous learning, and developed a questionnaire to measure learning families through an open-ended questionnaire survey. In Study 2, a four-dimensional questionnaire containing 16 items was obtained via exploratory factor analysis. Through confirmatory factor analysis and a discriminant validity test, Study 3 verified that the learning family scale and its four trait dimensions are differentiated in terms of content and structure. Study 4 examined the effect of learning families on individuals’ different behaviors based on different application contexts using experimental methods and questionnaires in cross-population samples, and the results showed that the learning family has a significant and positive effect on individuals’ creativity, innovative behaviors, prosocial behaviors, proactive behaviors, job performance, academic achievement and science literacy. Future research can further explore the effect mechanism underlying learning families on individual behaviors, thereby better explaining the process by which learning families influence individual behaviors.

### 6.1. Theoretical Contributions

This study makes three main theoretical contributions. First, this study used the bioecological system theory as a conceptual framework for the initial exploration of the construct of the learning family, clarified the concept of the learning family and its four characteristic dimensions, expanded the application boundary of the bioecological system theory, and enriched the theoretical foundation of the learning family. Most previous studies have explored the effects of microsystem, mesosystem, exosystem, and macrosystem environments on individuals’ development based on Bronfenbrenner’s early theories [12,13], ignoring the importance of individuals and time in the process of theoretical evolution. Moreover, few studies have applied the PPCT model of the bioecological system theory to the family field. Since no scholars have yet explored the inner structure of the learning family, based on the PPCT model of the bioecological system theory, this study regarded the learning family as a family ecosystem and systematically analyzed the key factors affecting individuals’ perceptions of the learning family from four characteristic dimensions—learning involvement, learning effectiveness, learning support, and continuous learning—which provides new theoretical support for an in-depth understanding of the concept and structure of the learning family.

Second, this study provides a reliable and valid tool for measuring individuals’ perceptions of the learning family. From qualitative to quantitative, this study examined and validated the concepts of the learning family and its characteristic dimensions, providing a new empirical basis for this for the first time. Most of the previous studies on the learning family are abstract and descriptive [15,19], lacking normative empirical testing, especially since there is still a gap in the research on the implementation of the learning family by combining qualitative and quantitative methods. In this study, a learning family scale was developed using normative methods, and there was a significant and positive correlation between each of the four characterization dimensions of the scale and their overall scores, reflecting the interaction between the learning family and its different dimensions. On the one hand, learning involvement is the basis for family members to acquire knowledge and skills, which can provide strong support for learning effectiveness, while increased learning effectiveness helps to create a strong family learning atmosphere and further enhances their learning ability. On the other hand, good learning support can provide family members with abundant learning resources and opportunities, prompting them to show continuity and motivation in the learning process, thus enhancing their perception of the learning family.

Third, this study reveals the stability of the learning family scale in cross-contextual situations based on different application contexts. Most previous studies on how family factors influence individual development have focused only on a single context or group [34,58]. In contrast, Study 4 examined the relationship between perceptions of the learning family and their behaviors among three groups: adolescents, college students, and on-the-job social personnel. The results showed that there was a significant positive correlation between the learning family and academic performance, science literacy, and prosocial behavior in the sample of adolescents. In the sample of college students, there was a significant positive correlation between the learning family and students’ creativity, innovative behavior, proactive behavior and prosocial behavior. In the sample of on-the-job social personnel, there was a significant positive correlation between the learning family and their creativity, innovation behavior, proactive behavior and job performance. As noted in previous studies, the influence of learning families on an individual’s study habits, divergent thinking style, and spirit of scientific inquiry is an important factor in enhancing their creativity [16]. Moreover, the results of this study also indicate that the learning family scale is effective across populations and across contexts and also reflects that the learning family will have an impact on the behavioral performance of individuals at different stages of their development, which lays the foundation for subsequent empirical research on the learning family.

### 6.2. Practical Implications

This study also has important practical implications. First, it provides a guiding path for constructing learning families. On the one hand, at the level of learning culture, this study explored the concept, structure and specific manifestations of the learning family, thus helping promote the shaping of family learning culture as well as the transformation of more families into learning families. Moreover, when an increasing number of families are transformed into learning families, this positive learning culture can gradually penetrate the social level and promote the improvement of the overall learning trend in society. On the other hand, at the level of family learning, this study analyzed the characteristic dimensions of the learning family and its specific manifestations, thereby providing direction and motivation for learning exchanges among family members, stimulating common learning on the part of family members, and promoting the generation of family learning behaviors. Moreover, it can help guide family members to adopt strategies such as optimizing learning involvement, enhancing learning effectiveness, strengthening learning support and encouraging continuous learning, thereby providing a reference for improving the comprehensive literacy of family members.

Second, this study also provides a management basis for the related organizations to improve the behavioral performance of individuals. This study revealed that the learning family was significantly and positively associated with individual creativity, innovative behaviors, prosocial behaviors, proactive behaviors, job performance, academic achievement, and science literacy, which suggests that the learning family can be used to identify new aspects of family influences that managers may not be aware of and could become a useful supplement to promote individual and organizational development. Specifically, from the perspective of educational management, the concept of the learning family can be introduced into family education guidance services, publicity and education activities related to the learning family can be conducted, the construction of learning families through home-school contacts can be promoted, and the contribution of the learning family to the growth of students can be improved. From the perspective of organizational management, managers should pay attention to the influence of family factors on employees’ behavioral performance, and through training and development, they can help employees better utilize the advantages of the learning family and improve their behavioral performance while improving the knowledge and skills of family members, thus promoting the sustainable development of individuals and organizations.

### 6.3. Limitations and Future Directions

This study also has some shortcomings that need to be addressed in future studies. First, the on-the-job social personnel in Study 4 only used their self-reported data and lacked multisource evaluation. In the future, we can explore the impact of the learning family on employee behavior using measures of leader–employee paired or team mutual evaluation. Additionally, large-scale surveys can be used in the future to further examine the differences in learning families among different groups and regions, and to explore their influencing factors, thus better generalizing the results of the study. Second, this study only verified the effect of the learning family on individual behavioral performance. In the future, we can further examine the association between the learning family and antecedent variables such as the individual growth environment, educational background, and social roles, as well as its mediating mechanisms and boundary conditions on individual behavior. Third, this study explored and validated the concept of the learning family and its characteristic dimensions in the Chinese context, and cross-cultural studies can be conducted in the future to further validate the cross-cultural adaptability of the learning family. Additionally, tracking data can be collected in the future to explore the development trend of the learning family and its dynamic association with individual behavior, and the inclusion of additional longitudinal studies or experimental designs can strengthen causal interpretation and demonstrate the lasting effects of a learning family environment over time.

## Figures and Tables

**Table 1:** Retained items after exploratory factor analysis.

Items	Learning Involvement	Learning Effectiveness	Learning Support	Continuous Learning	Commonality
1. My family members value righting their respective attitudes towards learning.	0.78				0.57
2. My family members value improving their respective learning abilities.	0.69				0.58
3. My family members often recognize and encourage my progress.	0.66				0.68
4. My family members can communicate ideas about learning equally.	0.65				0.66
5. The knowledge and experience that my family shares with me helps to enhance my contribution to the progress of society.		0.71			0.60
6. The knowledge and experience that my family shares with me helps to enhance my contribution to the development of the organization.		0.75			0.62
7. The knowledge and experience that my family shares with me helps to improve my knowledge level and learning ability.		0.53			0.45
8. The knowledge and experience that my family shares with me helps me to create and implement new ideas.		0.56			0.57
9. My family can provide opportunities for my learning.			0.77		0.69
10. My family can provide resources for my learning.			0.80		0.73
11. My family members are good at discovering new trends and opportunities for learning.			0.64		0.68
12. My family members are good at developing and learning a variety of new knowledge and skills.			0.52		0.65
13. My family members encourage me to keep learning and updating my knowledge and skills.				0.75	0.64
14. My family members encourage me to keep learning and improving my abilities and qualifications.				0.74	0.60
15. My family members encourage me to face current learning difficulties correctly.				0.64	0.54
16. My family members encourage me to face future learning challenges correctly.				0.57	0.55
Eigenvalues	6.03	1.63	1.12	1.02	
Percentage of variance	37.71%	10.18%	7.01%	6.40%	61.30%
Cronbach’α	0.79	0.74	0.82	0.70	0.95

**Table 2:** Results of the confirmatory factor analysis.

Models	*χ* ^2^	*df*	*χ*^2^/*df*	CFI	TLI	RMSEA	SRMR	Standardized Loading Min.	AVE Min.
Four-factor model	395.55 ***	98	4.04	0.92	0.90	0.08	0.05	0.63	0.51
Three-factor model	475.21 ***	101	4.71	0.90	0.88	0.09	0.05	0.63	0.49
Two-factor model	660.70 ***	103	6.41	0.85	0.83	0.11	0.06	0.62	0.44
One-factor model	775.45 ***	104	7.46	0.82	0.80	0.12	0.07	0.60	0.43

Note: *** *p* < 0.001. Four-factor model: learning involvement, learning effectiveness, learning support, continuous learning; three-factor model: learning involvement + learning effectiveness, learning support, continuous learning; two-factor model: learning involvement + learning effectiveness, learning support + continuous learning; one-factor model: learning involvement + learning effectiveness + learning support + continuous learning. “+” stands for two factors combined into one factor.

**Table 3:** The correlation coefficient between the factors of learning family scale.

Variable	*M*	*SD*	1	2	3	4
1. Learning effectiveness	5.39	0.99	(0.73)			
2. Learning involvement	5.47	1.06	0.67 **	(0.74)		
3. Learning support	5.21	1.05	0.61 **	0.68 **	(0.71)	
4. Continuous learning	5.76	0.92	0.55 **	0.63 **	0.58 **	(0.74)
5. Total learning family score	5.46	0.85	0.84 **	0.88 **	0.85 **	0.80 **

Note: ** *p* < 0.01. The values in parentheses are AVE square root values.

**Table 4:** Results of the discriminant validity analysis.

Models	*χ* ^2^	*df*	*χ*^2^/*df*	CFI	TLI	RMSEA	SRMR
Two-factor model (learning family; parental parenting style)	40.29 ***	17	2.37	0.98	0.96	0.08	0.02
One-factor model (learning family + parental parenting style)	213.90 ***	20	10.70	0.80	0.72	0.21	0.07
Two-factor model (learning family; parental involvement)	17.85 ***	13	1.37	0.99	0.99	0.04	0.02
One-factor model (learning family + parental involvement)	191.37 ***	14	13.67	0.66	0.49	0.24	0.37
Two-factor model (learning family; family functioning)	94.25 ***	39	2.42	0.97	0.95	0.08	0.03
One-factor model (learning family + family functioning)	258.84 ***	44	5.88	0.87	0.84	0.15	0.04
Two-factor model (learning family; learning organization)	41.57 ***	26	1.60	0.99	0.98	0.05	0.02
One-factor model (learning family + learning organization)	128.26 ***	27	4.75	0.92	0.89	0.13	0.04

Note: *** *p* < 0.001. “+” stands for two factors combined into one factor.

**Table 5:** Results of confirmatory factor analysis in Sample 2.

Models	*χ* ^2^	*df*	*χ*^2^/*df*	CFI	TLI	RMSEA	SRMR
Five-factor model (LF; IB; C; PB; JP)	549.70 ***	289	1.90	0.91	0.90	0.07	0.05
Four-factor model (LF; IB + C; PB; JP)	579.78 ***	293	1.98	0.90	0.89	0.07	0.05
Three-factor model (LF; IB + C; PB + JP)	631.03 ***	296	2.13	0.88	0.87	0.08	0.06
Two-factor model (LF; IB + C + PB + JP)	635.16 ***	298	2.13	0.87	0.87	0.08	0.06
One-factor model (LF + IB + C + PB + JP)	745.42 ***	299	2.49	0.84	0.83	0.09	0.06

Note: LF = learning family; IB = innovative behavior; C = creativity; PB = proactive behavior; JP = job performance; “+” stands for two factors combined into one factor. *** *p* < 0.001.

**Table 6:** Results of confirmatory factor analysis in Sample 3.

Models	*χ* ^2^	*df*	*χ*^2^/*df*	CFI	TLI	RMSEA	SRMR
Three-factor model (LF; SL; PB)	82.65 ***	62	1.33	0.99	0.99	0.05	0.04
Two-factor model (LF; SL + PB)	408.64 ***	64	6.39	0.80	0.76	0.19	0.11
One-factor model (LF + SL + PB)	595.79 ***	65	9.17	0.69	0.63	0.24	0.15

Note: LF = learning family; SL = science literacy; PB = proactive behavior; “+” stands for two factors combined into one factor. *** *p* < 0.001.

**Table 7:** Means, standard deviations, and correlation coefficients of variables in Sample 2.

Variables	*M*	*SD*	1	2	3	4	5	6	7	8	9	10
1. Gender	0.30	0.46										
2. Age	31.88	5.63	0.06									
3. Education	3.30	0.52	−0.18 **	0.07								
4. Job tenure	7.57	5.11	0.09	0.90 **	−0.07							
5. Self-educational expectation	5.71	0.61	−0.25 **	0.06	0.50 **	−0.07						
6. Family socioeconomic status	0.00	4.03	−0.21 **	0.01	0.27 **	−0.07	0.21 **					
7. Learning family	5.85	0.61	−0.00	0.12	0.13	0.03	−0.01	0.33 **				
8. Innovative behavior	5.81	0.74	0.03	0.03	0.16 *	−0.05	0.09	0.24 **	0.70 **			
9. Creativity	5.64	0.95	−0.11	0.01	0.21 **	−0.09	0.07	0.29 **	0.74 **	0.87 **		
10. Proactive behavior	5.78	0.71	−0.02	0.13	0.11	0.05	0.05	0.23 **	0.69 **	0.88 **	0.82 **	
11. Job performance	5.91	0.44	0.17	0.14 *	−0.03	0.10	−0.15 *	0.12	0.67 **	0.67 **	0.60 **	0.67 **

Note: *n* = 200, ** *p* < 0.01, * *p* < 0.05.

**Table 8:** Means, standard deviations and correlation coefficients of variables in Sample 3.

Variables	*M*	*SD*	1	2	3	4	5	6	7	8
1. Gender	0.47	0.50								
2. Age	13.78	2.32	0.07							
3. Grade	7.68	2.42	0.00	0.95 **						
4. Parental educational expectation	6.37	5.16	−0.10	0.02	0.01					
5. Family socioeconomic status	0.00	3.72	−0.02	−0.22 **	−0.18 *	0.11				
6. Learning family	5.81	0.70	−0.06	−0.05	−0.05	0.16	0.03			
7. Science literacy	5.00	0.89	0.02	0.22 **	0.20 *	0.05	−0.22 **	0.41 **		
8. Prosocial behavior	5.88	1.00	−0.08	0.10	0.07	0.05	−0.31 **	0.23 **	0.67 **	
9. Academic achievement	0.00	2.57	−0.05	−0.02	−0.00	0.05	0.13	0.18 *	0.36 **	0.15

Note: *n* = 145, ** *p* < 0.01, * *p* < 0.05.

**Table 9:** Regression analysis results for Sample 1.

Variables	Innovative Behaviors		Creativity		Prosocial Behaviors		Proactive Behaviors	
	M1	M2	M3	M4	M5	M6	M7	M8
Gender	0.01	0.02	0.04	0.04	−0.03	−0.02	0.05	0.06
Age	0.06	0.04	0.02	−0.00	−0.00	−0.02	0.02	0.00
Education	0.06	−0.01	0.07	0.00	0.05	−0.02	0.05	−0.02
Self-educational expectation	−0.13	−0.05	−0.11	−0.03	−0.07	0.00	−0.07	0.00
Learning family		0.86 ***		0.87 ***		0.85 ***		0.82 ***
R^2^	0.02	0.76	0.02	0.77	0.01	0.72	0.01	0.68
F	1.00	119.56 ***	0.73	129.75 ***	0.26	100.18 ***	0.45	80.94 ***

Note: The data in this table are standardized regression coefficients. *** *p* < 0.001.

**Table 10:** Regression analysis results for Sample 2.

Variables	Innovative Behaviors		Creativity		Proactive Behaviors		Job Performance	
	M1	M2	M3	M4	M5	M6	M7	M8
Gender	0.10	0.06	−0.05	−0.08	0.02	−0.01	0.16 *	0.12 *
Age	0.27	−0.00	0.37 *	0.10	0.35 *	0.09	0.32	0.06
Education	0.09	0.03	0.14	0.09	0.03	−0.02	0.01	−0.04
Job tenure	−0.28	−0.07	−0.41 *	−0.19	−0.26	−0.05	−0.20	0.00
Self-educational expectation	−0.01	0.09	−0.12	−0.01	−0.04	0.06	−0.19 *	−0.09
Family socioeconomic status	0.22 **	−0.01	0.23 **	0.00	0.21 **	−0.01	0.17 *	−0.05
Learning family		0.70 ***		0.72 ***		0.69 ***		0.68 ***
R^2^	0.09	0.51	0.14	0.57	0.08	0.48	0.10	0.49
F	3.21 **	28.56 ***	5.08 ***	36.95 ***	2.83 *	25.61 ***	3.50 **	26.47 ***

Note: The data in this table are standardized regression coefficients. *** *p* < 0.001, ** *p* < 0.01, * *p* < 0.05.

**Table 11:** Regression analysis results for Sample 3.

Variables	Science Literacy		Prosocial Behaviors		Academic Achievement	
	M1	M2	M3	M4	M5	M6
Gender	0.01	0.03	−0.09	−0.08	−0.04	−0.03
Age	0.26	0.26	0.27	0.27	−0.09	−0.09
Grade	−0.08	−0.06	−0.24	−0.23	0.11	0.12
Parental educational expectation	0.07	0.01	0.07	0.04	0.03	0.01
Family socioeconomic status	−0.18 *	−0.18 *	−0.30 ***	−0.30 ***	0.13	0.13
Learning family		0.42 ***		0.23 **		0.18 *
R^2^	0.09	0.26	0.11	0.16	0.02	0.05
F	2.60 *	7.99 ***	3.55 **	4.51 ***	0.62	1.28

Note: The data in the table are standardized regression coefficients. *** *p* < 0.001, ** *p* < 0.01, * *p* < 0.05.

## Data Availability

The data that support the findings of this study are available upon request from the corresponding author.
